# The Jarisch-Herxheimer Reaction in Leptospirosis: A Systematic Review

**DOI:** 10.1371/journal.pone.0059266

**Published:** 2013-03-26

**Authors:** Gilles Guerrier, Eric D’Ortenzio

**Affiliations:** 1 Département d’Anesthésie-Réanimation, Centre Hospitalier Territorial, Noumea, Nouvelle Calédonie; 2 Unité d’Epidémiologie des Maladies Infectieuses, Institut Pasteur de Nouvelle-Calédonie, Réseau International des Instituts Pasteur, Noumea, Nouvelle Calédonie; Federal University of Pelotas, Brazil

## Abstract

**Background:**

Leptospirosis is an endemo-epidemic zoonotic disease associated with potentially fatal renal, cardiovascular or pulmonary failure. Recommended treatment includes antibiotics, which may induce a Jarisch-Herxheimer reaction (JHR). Since little information on the importance of this adverse event is available, we performed this review to quantify frequency and impact of JHR in leptospirosis management.

**Methodology/Principal Findings:**

This review systematically summarizes the literature on the JHR in leptospirosis. To approach the broader aspects of the subject, articles considering the treatment of leptospirosis, national leptospirosis guidelines and textbook and technical reports of the World Health Organisation were reviewed. Publications describing JHR in leptospirosis are very limited and consist mainly of single case reports and small case series. A single randomized control trial specifically assessed the JHR occurrence, but it has never been systematically investigated in large trials. Not all guidelines and not all literature on leptospirosis mention this reaction which can be fatal.

**Conclusions/Significance:**

Although generally assumed to be a rare event, the true prevalence of JHR in leptospirosis is unknown and the awareness of this event is insufficient. All leptospirosis guidelines and local leptospirosis protocols should stress on systematic monitoring for clinical status early after antibiotic administration. Large well designed studies are required to precise the incidence and the impact of JHR as well as the severity and rates between various antibiotics.

## Introduction

Leptospirosis is a widespread zoonotic disease of worldwide distribution caused by pathogenic *Leptospira*, a spirochetal organism that is transmitted to humans by exposure to urine of infected mammalian reservoirs such as rodents or wild and domestic animals [1]. The disease has a wide-ranging clinical spectrum from asymptomatic forms to severe presentations. The latter are estimated to occur in 5–15% of all human infections and commonly combine jaundice, renal failure, myocarditis and/or haemorrhage [2] with a significant mortality rate. While a single experimental study demonstrated the relevance of antibiotic administration even in late stages of leptopsirosis [3] the benefit of antibiotic therapy on mortality lacks of clinical evidence [4]. However, the current treatment guidelines still rely on antibiotic administration regardless of stage or severity of the disease [5]. Initiation of chemotherapy in spirochetal diseases may precipitate a febrile inflammatory reaction [6], [7], known as the Jarisch-Herxheimer reaction (JHR), originally described in patients with syphilis receiving mercury treatment [8],[9]. This reaction is characterised by an acute inflammatory response associated with the release of large amounts of cytokines, resulting from clearance of spirochetes from the circulation [6], [7]. Prevalence, clinical manifestations and outcome of JHR have been well studied in syphilis [10], Lyme disease [11], tick-born relapsing fever [12] and louse-born relapsing fever [13]–[15]. However, a clear idea of importance JHR in leptospirosis is lacking. In order to quantify frequency and impact of JHR in leptospirosis, we systematically reviewed the published literature and put it in a broader perspective, identifying data gaps that should be addressed.

## Methods

### Search Strategy and Selection Criteria

The inclusion criteria were as follows: all publications on leptospirosis treated with antibiotics providing information about the occurrence of early adverse events including JHR were selected. When the original article was not obtainable but the abstract containing the requested information was, the publication was included in the analysis. To avoid multiple counting (duplication) of identical procedures and cases, follow-up publications on identical procedures and cases were traced and excluded. The electronic database PubMed was searched with the keywords “leptospirosis” and “Leptospira”. A second search was performed for “penicillin”, “amoxicillin”, “ampicillin”, “cefepime”, “ceftriaxone” and “antibiotics”. A final search was done with “Jarisch-Herxheimer reaction”. Search results were assessed using different combinations. The search was not limited by study design, language or date of publication. A similar search was conducted with Embase. The Cochrane Library was also searched for leptospirosis publications. World Health Organisation (WHO) websites and international or accessible national guidelines in endemic countries were searched, and the latest editions of five standard textbooks in infectious and tropical diseases were studied [16]–[20]. Titles and abstracts were screened, and potentially relevant case reports, case series and trials were further evaluated. Reports were included if they assessed the occurrence of JHR in leptospirosis. Reference lists of included articles were screened for further relevant publications. Study identification and data extraction were conducted independently and cross-checked for accuracy by both authors.

### Data Abstraction and Methodological Assessment

The primary outcome was JHR occurrence or rates measured at time points described by investigators. Secondary outcomes include renal failure rates and mortality. Serovars were also recorded. It was impossible to retrospectively grade the severity of the JHR due to the lack of a standardized definition of the events.

## Results

Twenty eight publications met our inclusion criteria, with a total number of 976 leptospirosis cases treated with antibiotics. One publication was a follow-up report on identical case series and therefore excluded from analysis [21]. Abstract or full text was not found for an additional publication which was also excluded [22]. One previous review dedicated to the topic was published in 1991 without systematically assessing the literature [23]. Among the selected studies, 11 patients presenting with concomitant infections were excluded [24].

### Studies Characteristics and JHR Occurrence

Eight case reports [25]–[32] and five case series [23], [33]–[36] published between 1955 and 2012 were reviewed and analyzed. All selected patients had a laboratory confirmed leptospirosis without concomitant infection and all were treated with antibiotics. A JHR was reported in 92 patients from 1 to 48 hours after administration of the first dose of antibiotics. The nature of antibiotics regimen given varied considerably: penicillin (n = 81), ampicillin (n = 8), ceftriaxone (n = 1), cefepime (n = 1) and penicillin and ceftriaxone (n = 1). The most common features of described JHR were sudden onset of shivering or rigors (n = 6), with rise in temperature (n = 9), with (n = 8) or without (n = 1) a fall in blood pressure, occurring after administration of the first dose of antibiotics (Table 1). The largest case series accounted for 70 cases, out of 84 case-patients treated with penicillin in Malaya in 1957 [36].

**Table 1:** Case reports and case series of Jarisch-Heirxheimer reaction after admistration of antibiotics for the treatment of leptospirosis.

References	Country	Studydesign	Treatmentadministered	Delaybefore JHR	Symptoms	Outcome
Lau CL et al.	American Samoa	Case report	penicillin	1 hour	Increase in fever	Discharged
Emerg Infect Dis		(n = 1)			Rigors	
2012					Severe Headache	
Markham R et al.	Australia	Case report	benzylpenicillin and	2 hours	Tachycardia	Discharged
Med J Aust		(n = 1)	ceftriaxone		Tachypnea	
2012					Hypertension	
					Severe rigors	
Hashimoto T et al.	Japan	Case report	ceftriaxone	2 days	Pulmonary deterioration	Discharged
J Thorac Imaging		(n = 1)				
2012						
Masuda K et al.	Japan	Case report	cefepime	Unknown	Chills	Discharged
Kansenshogaku Zasshi		(n = 1)			Fever	
2010					Hypotension	
Narita M et al.	Japan	Case series	ampicillin (n = 6)	Unknown	Rigors	Unknown
Am J Trop Med Hyg		(n = 6)			Hypotension	
2005						
Tattevin P et al.	France	Case report	amoxicillin	4 hours	Headache	Discharged
Eur J Clin Microbiol Infect Dis		(n = 1)			Hypotension	
2003					Tachycardia	
					Fever	
					Nuchal rigidity	
Swiader L et al.	France	Case report	penicillin	8 hours	Headache	Unknown
La Presse Med		(n = 1)			Photophobia	
1995					Nuchal rigidity	
Vaughan C et al.	Ireland	Case series	benzylpenicillin (n = 2)	4 to 5 hours	Increase in fever	Died (n = 1)
Postgrad Med J		(n = 3)	ampicillin (n = 1)		Hypotension	Discharged (n = 2)
1994						
Emmanouilides CE et al.	USA	Case report	penicillin	Few hours	Fever	Discharged
Clin Infect Dis		(n = 1)			Chills	
1991					Hypotension	
					Respiratory distress	
Friedland JS et al.	UK	Case series	benzylpenicillin (n = 2)	5 hours	Fever	Discharged
Rev Infect Dis		(n = 2)		4 hours	Severe rigor	
1991					Hypotension	
					Abdominal pain	
					Headache	
					Fever	
					Profuse vomiting	
Winearls CG et al.	UK	Cases series	penicillin (n = 3)	Unknown	Sharp rise in temperature	Discharged
Q J Med		(n = 3)			Rigor	
1984						
Mackay-Dick J et al.	Malaysia	Case series	penicillin (n = 70)	Unknown	Fever (n = 59)	Discharged
J Royal Army Medical Corps		(n = 70)			Aggravation of classicalsymptoms (n = 31)	
1957					Hypotension (n = 58)	
					Oligo-anuric (all)	
Crooks J et al.	Scotland	Case report	penicillin	2 ½ hours	Rigor	Discharged
Br Med J		(n = 1)			Fever	
1955					Weakness	
					Low blood pressure	

Seven randomized control trials (RCT) [21], [24], [37]–[42], six non randomized control trials (NRCT) [43]–[48] were reviewed and analyzed (Table 2). With the exception of two RCTs [41], [42], all studies monitoring the efficacy of antibiotics were not designed to assess adverse events linked to antibiotics including the JHR occurrence. The single study designed to monitor adverse events focusing on leptospirosis-related JHR was performed in 1986 by Watt et al. [42]. In this study, a single Herxheimer-like reaction was observed in a patient receiving saline placebo. The authors concluded that fear of a Herxheimer-like reaction should not dissuade clinicians from administering antibiotics to patients with leptospirosis. However, studied patients received up to four doses of parenteral antibiotic or had completed up to two days of an oral antibiotic regimen before inclusion.

**Table 2:** Clinical trials for leptospirosis treatment in which adverse events were searched.

References	Country Yearof study	Study design	Treatment	Any adverse events reported	Systematic assessment of JHR	Observationof JHR	Delaybefore JHR	Symptoms of JHR	Outcome
Phimda K et al.	Thailand	RCT	doxycycline (n = 34)	Rash	Unknown	No	NA	NA	No death
Antimicrob Agents Chemother	2003–2005		vs	Nausea					
2007			azythromycin (n = 35)	Vomiting					
				Diarrhea					
				Abdominal pain					
				Dizziness					
Suputtamongkol Y et al.	Thailand	RCT	penicillin (n = 52)	Skin rash	Unknown	No	NA	NA	4 deaths
Clin Infect Dis	2001–2002		vs						
2004			cefotaxime (n = 59)						
			vs						
			doxycycline (n = 53)						
Panaphut T et al.	Thailand	RCT	ceftriaxone (n = 87)	No	Unknown	No	NA	NA	10 deaths
Clin Infect Dis	2000–2001		vs						
2003			penicillin (n = 86)						
Costa E et al.	Brazil	RCT	penicillin (n = 125)	No	Unknown	No	NA	NA	15 deaths
Rev Inst Med Trop Sao Paulo	1997–1999		vs						
2003			placebo (n = 128)						
Daher EdF et al.	Brazil	NRCT	penicillin (n = 16)	No	Yes	No	NA	NA	1 death
Rev Inst Med Trop Sao Paulo	1996–1998		vs						
2000			untreated (n = 19)						
Marotto PCF et al.	Brazil	NRCT	penicillin or ampicillin (n = 28)	No	Unknown	No	NA	NA	1 death
Am J Trop Med Hyg	1989–1995	(retrospective analysis of children)	vs						
1997			untreated (15)						
Watt G et al.	Philippines	RCT	penicillin ((n = 24)	No	Yes	Yes (n = 1 in patient receiving placebo)	Unknown	Unknown	No death
J Infect Dis	1985–1986		vs						
1990			placebo (n = 16)						
Edwards CN et al.	Barbados	RCT	penicillin (n = 38)	No	Unknown	Yes (n = 1 in patient receiving penicillin)	3 hours	Unknown	4 deaths
Am J Trop Med Hyg	1983–1986		vs						
1986			placebo (n = 41)						
McClain BL et al.	Panama	RCT	doxycycline (n = 14)	No	Yes	No	NA	NA	No death
Ann Intern Med	Year not specified		vs						
1984			placebo (n = 15)						
Russel RW et al.	Malaya	NRCT	oxytetracycline (n = 27)	Pyrexia	No	No	NA	NA	Unknown
Lancet	Year not specified		vs	Sore throat					
1958			placebo (n = 25)	Erythema					
				Urticaria					
Doherty RL et al.	Australia	NRCT	penicillin (n = 71)	No	No	No	NA	NA	Unknown
Australas annals of Medicine	Year not specified		vs						
1955			chloremphenicol (n = 12)						
			vs						
			chloremphenicol+penicillin (n = 20)						
Fairburn AC et al.	Malaya	NRCT	penicillin (n = 10)	No	No	No	NA	NA	No death
Lancet	Year not specified		vs						
1956			chloramphenicol (n = 14)						
			vs						
			placebo (n = 22)						
Hall HE et al.	Puerto Rico	NRCT	penicillin (n = 5)	No	No	No	NA	NA	2 deaths
Annals of Internal Medicine	Year not specified		vs						
1951			chloremphenicol (n = 18)						
			vs						
			aureomycin (n = 13)						
			vs						
			terramycin (n = 8)						
			vs						
			streptomycin (n = 12)						
			vs						
			aureomycin+streptomycin (n = 9)						
			vs						
			aureomycin+cortison (n = 2)						
			vs						
			untreated (n = 12)						

RCT: Randomized Control Trial; NRCT: Non Randomized Control Trial.

### JHR in National and International Guidelines, Textbooks and WHO/ILS Guideline

Among 3 screened national or international guidelines, all described JHR as a potential adverse event, although occurring with penicillin only [5], [49], [50]. Three textbooks out of the five consulted mentioned the possibility of JHR early after penicillin initiation [18]–[20].

### JHR Management and Outcome

JHR management was not specified in 82 cases, but reported with no details in five cases (supportive care). In the remaining cases, the management consisted in fluid infusion, vasopressors (n = 1), corticosteroids and inotropic support (n = 1) and transient dialysis (n = 1). Overall, eleven studies mentioned immediate outcome after JHR occurrence. Among them, all cases fully recovered with the exception of one JHR-related death [34].

### JHR and Leptospira Serovars

Out of 13 case reports or case series reporting JHR, the pathogen serovar was stated in 18 patients. Nine different types of serovar were involved in JHR-patients, the most prevalent being Icterohaemorrhagiae (n = 6).

## Discussion

To the best of our knowledge, this systematic review is the first to formally appraise the measurement of JHR occurrence in leptospirosis treated with antibiotics. Publications describing JHR consist mainly of single case reports or case series. All RCTs included in our review but one were not designed to assess JHR incidence and therefore judged to be of poor methodological quality. Moreover, methods used to monitor JHR were not described in enough detail to reach predefined criteria and to determine whether there was a link between JHR occurrence and organ failure or death. Most reports were targeted at criteria such as duration of fever, clearance of spirochetes and length of stay. The single appropriately designed and detailed RCT had major limitations, including a small number of subjects and a lack of JHR confirmation.

However, this work will help to better estimate the potentially detrimental impact of antibiotics in leptospirosis and inform future management guidelines. Since there is little doubt that renal damage can be aggravated by vascular hypotension, particular attention should be paid to monitor blood pressure after initiation of antibiotics in suspected leptospirosis, especially in particular conditions such as pregnant, hypotensive or chronic renal insufficiency presenting patients. Of note, JHR is not reported in studies describing prognostic factors of leptospirosis but clinicians are usually facing complicated management [51], [52].

Interestingly, a higher proportion of JHR occurred in early stage leptospirosis, suggesting a higher probability of the event before the natural clearance of spirochetes. It is postulated that the inflammatory process results from activation of the cytokine cascade during the degeneration of spirochetes. Therefore, the apparent lower proportion of JHR in patients with leptospirosis compared with patients diagnosed with other spirochetal diseases may be explained by a lower bacteraemia. This hypothesis requires further investigation for validation. JHR unobserved or unreported by clinicians is an additional reason for this ostensible reduced frequency. Conversely, a confusion of JHR symptoms with the aggravation of the leptospirosis itself may lead to overestimate the incidence of this event.

Interestingly, international guidelines for the management of leptospirosis briefly mention the occurrence of JHR with penicillin, omitting the details of its prevention, management or outcome. Moreover, antibiotics other than penicillin (e.g. ceftriaxone) are not evoked. Similarly, infectious diseases textbooks mentioning the event overlook this reaction with non-penicillin antibiotics. Noteworthy, recommended antibiotics for the management of leptospirosis vary considerably according to settings. For example, most commonly used drugs in the Philippines for mild leptospirosis is doxycyclin while penicillin G is recommended for treating severe cases [50]; amoxicillin and cefotaxim are preferentially used in French Caribbean or in New Caledonia in mild and severe cases, respectively [53].

Some limitations in the current review should be acknowledged. First, potential publication bias is impossible to completely exclude. Moreover, unpublished reports or reports that were not referenced in databases could have been missed despite the comprehensive search. Second, patients presenting with undetected co-infections may have been included in the study. Finally and most importantly, the diagnosis of JHR was not supported by any dosage of biological markers. In addition, the definition of JHR was not uniform, including in the largest case series published by Mackay-Dick [36] which lacks of details such as the onset of reaction. Therefore, although most of the cases presented a genuine JHR, some might have had a clinical aggravation related to the spirochetal disease regardless of the antibiotherapy.

### Conclusions

The prevalence of JHR in leptospirosis treated with antibiotics is unknown and the awareness of this adverse event is insufficient. Although this review suggests that antibiotic treatment in patients with leptospirosis may result in less common and less severe JHR than in patients with other spirochetal diseases, all leptospirosis guidelines and local leptospirosis protocols should stress on systematic monitoring for clinical status early after antibiotic administration. We strongly recommend that patients receiving penicillin or other antibiotics for the management of leptospirosis to be monitored early after initiation of treatment to prevent any detrimental effects of a potential JHR. Since it is still controversial whether antimicrobials produce a beneficial effect in mild human leptospirosis, large well designed studies are required integrating a specific monitoring to precise the incidence and the impact of JHR as well as the severity and rates between various antibiotics.
